# Rationally Engineering pH Adaptation of Acid‐Induced Arginine Decarboxylase from *Escherichia coli* to Alkaline Environments to Efficiently Biosynthesize Putrescine

**DOI:** 10.1002/advs.202307779

**Published:** 2024-04-03

**Authors:** Li Wang, Bo Ding, Xiangyang Hu, Guohui Li, Yu Deng

**Affiliations:** ^1^ National Engineering Research Center of Cereal Fermentation and Food Biomanufacturing Jiangsu Provincial Research Center for Bioactive Product Processing Technology Jiangnan University 1800 Lihu Road Wuxi Jiangsu 214122 China

**Keywords:** arginine decarboxylase, biocatalysis, pH adaptation, protein engineering, putrescine

## Abstract

Acid‐induced arginine decarboxylase AdiA is a typical homo‐oligomeric protein biosynthesizing alkaline nylon monomer putrescine. However, upon loss of the AdiA decamer oligomeric state at neutral and alkaline conditions the activity also diminishes, obstructing the whole‐cell biosynthesis of alkaline putrescine. Here, a structure cohesion strategy is proposed to change the pH adaptation of AdiA to alkaline environments based on the rational engineering of meridional and latitudinal oligomerization interfaces. After integrating substitutions of E467K at the latitudinal interface and H736E at the meridional channel interface, the structural stability of AdiA decamer and its substrate transport efficiency at neutral and alkaline conditions are improved. Finally, E467K_H736E is well adapted to neutral and alkaline environments (pH 7.0–9.0), and its enzymatic activity is 35‐fold higher than that of wild AdiA at pH 8.0. Using E467K_H736E in the putrescine synthesis pathway, the titer of putrescine is up to 128.9 g·L^−1^ with a conversion of 0.94 mol·mol^−1^ in whole‐cell catalysis. Additionally, the neutral pH adaptation of lysine decarboxylase, with a decamer structure similar to AdiA, is also improved using this cohesion strategy, providing an option for pH‐adaptation engineering of other oligomeric decarboxylases.

## Graphical abstract

The cohesion of decameric oligomerization of acid‐inducible arginine decarboxylase AdiA is enhanced by rational engineering at the meridional and latitudinal oligomerization interfaces. Ultimately, the pH adaptation of AdiA‐M2 to neutral and alkaline environments is significantly improved. Alkaline putrescine is efficiently biosynthesized with a titer of 128.9 g·L^–1^ and a conversion rate of 0.94 mol·mol^–1^ using AdiA‐M2.

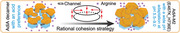

## 1 Introduction

Protein homo‐oligomerization is a common property that develops during natural evolution, with about half of proteins existing as homo‐oligomers.^[^ ^1^ ^]^ Homo‐oligomeric proteins are widely used in physiological regulation, medicine, and chemicals, such as alkaline amino acid decarboxylase, tyrosyl‐tRNA synthetase, and G protein‐coupled receptors.^[^ ^2^, ^3^, ^4^, ^5^, ^6^ ^]^ Intracellular homo‐oligomeric proteins are dominated by even‐numbered oligomeric dimers and tetramers that exhibit a dual‐axis symmetry. In contrast, high‐order oligomeric proteins are sparse.^[^ ^7^ ^]^ The oligomerization is essential for their functional activity and structural stability,^[^ ^8^ ^]^ which occurs through covalent or weak bonds between the monomers, controlled by several key interacting residues at the interface.^[^ ^9^ ^]^ This oligomerization is a dynamic process, regulated by protein concentration, ionic strength, pH, and cofactors, which has significant implications for controlling the biological activity of proteins.^[^ ^10^ ^]^ However, in industrial applications, optimal catalytic conditions for oligomeric proteins may conflict with their spontaneous oligomerization conditions. Therefore, various engineering strategies, based on key amino acid residues in interfacial interactions, have been proposed to artificially promote protein oligomerization, such as regulating interfacial residue complementarity, introducing disulfide bonds or electrostatic attraction, and improving interfacial hydrophobicity.^[^ ^11^ ^]^

**Figure 1:**
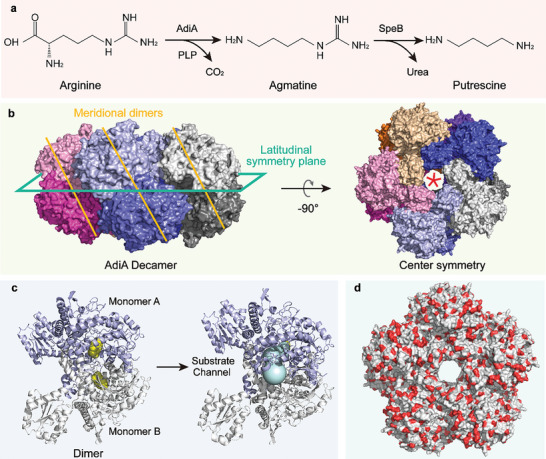
Structure of arginine decarboxylase. a) Conversion of arginine to agmatine by AdiA. b) Meridional and latitudinal views of the AdiA decamer 3D structure (PDB ID: 2vyc). c) Active pocket and substrate channel located within the AdiA dimer. In the dimer, the structure colored purple is monomer A, and the structure colored gray is monomer B. The structure colored yellow represents the cofactor pyridoxal phosphate (PLP) located at the active center. d) Distribution of acidic amino acids on the surface of AdiA. Acidic amino acids are colored red.

Artificial oligomerization of dimers and tetramers is readily achievable.^[^ ^10^ ^]^ However, for high‐order oligomers, this process is challenging due to the complex interfacial interactions and difficulties in molecular dynamics simulations. Arginine decarboxylase AdiA, a key enzyme for synthesizing alkaline putrescine (**Figure** **1**a), is a typical high‐order homo‐oligomeric protein (a decamer).^[^ ^12^ ^]^ This decamer is composed of five dimers forming bilayer pentameric rings, with a centrosymmetric structure (Figure 1b).^[^ ^12^ ^]^ The structure of the AdiA decamer can be split into two perspectives: meridional and latitudinal (Figure 1b). The meridional direction includes five dimers formed by the cross and interlock of two monomers, and the latitudinal direction includes two pentameric rings formed by five monomers interacting with each other head to tail. Accordingly, the oligomerization interface of AdiA decamer involves two distinct aspects: i) latitudinal interface between bilayer pentameric rings, that is, within dimers, and ii) meridional interface between monomers in the pentameric ring.

At the latitudinal interface, two centrosymmetric active pockets and substrate channels are formed in one dimer (Figure 1c). Because of the loose structure of the individual dimers, these dimers can function well for arginine decarboxylation only when they form a bilayer cyclic decamer. However, the oligomerization of AdiA decamer is reversibly regulated by pH.^[^ ^12^ ^]^ Due to the large amount of acidic amino acids on the surface of AdiA (Figure 1d), the decamer is only stable under acidic conditions (pH < 6.0) where the chargeability of acidic amino acids can be neutralized by protons.^[^ ^12^ ^]^ This acid activation phenomenon makes AdiA an important member of the acid stress response system of *Escherichia coli* (*E. coli*), ensuring cell survival in an acidic environment. In neutral and alkaline conditions, the activity of AdiA will be inhibited for its presence as a dimer or monomer.^[^ ^12^, ^13^, ^14^ ^]^ This pH inhibition effect is detrimental to the biosynthesis of alkaline putrescine in whole‐cell systems. In addition, lysine decarboxylases CadA, another important member in the acid stress response system, possesses a pH‐regulated mechanism for the oligomerization of bilayer cyclic decamers similar to that of AdiA.^[^ ^15^, ^16^ ^]^ The interfacial analysis theory of AdiA may be applicable to CadA to engineer its pH adaptation.

In this study, we rationally proposed a cohesion strategy for high‐order oligomeric proteins. This strategy included the engineering of electrostatic repulsion at the meridional interface between pentameric ring monomers, and substrate affinity and monomer compactness at the latitudinal channel interface, by exploiting the electrostatic affinity between acidic and basic amino acids. A high‐order decameric AdiA, with a stable oligomerization structure under neutral and alkaline conditions, was obtained using this engineered strategy, which could be used for the efficient biosynthesis of putrescine. Furthermore, the cohesion strategy also has the potential to engineer the oligomeric structure stability of the similar oligomeric protein CadA.

## 2 Results and Discussion

### 2.1 Rationally Cohesion Engineering pH Adaptation of AdiA to Alkaline

**Figure 2:**
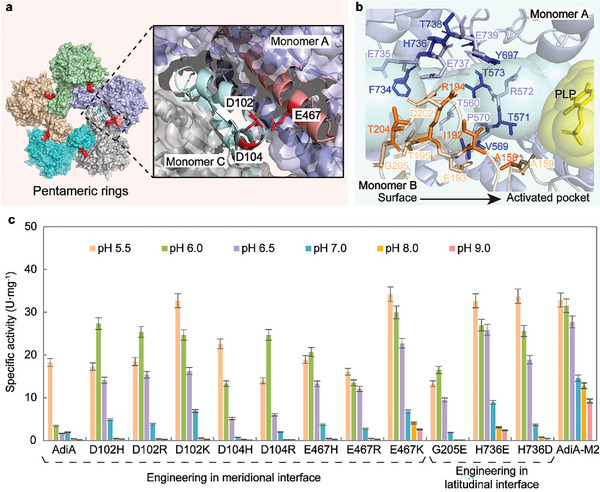
Rational engineering of AdiA based on oligomerization structural stability and substrate channel. a) Acidic amino acid enriched regions at the interface in the pentameric ring (colored red). The α‐helix colored salmon and E467 belonged to monomer A. The α‐helix colored blue, and D102 and D104 belonged to monomer C. b) Substrate channel of AdiA. The blue and purple labeled residues were from monomer A; the yellow labeled residues are from monomer B. c) Enzymatic activity of AdiA variants at the interface of the pentameric ring, the substrate channel, and E467K_H736E (AdiA‐M2). Enzymatic activity was determined in 1/15 mol·L^−1^ sodium–potassium phosphate buffer (pH 5.5, 6.0, 6.5 or 7.0) or Tris‐HCl buffer (pH 8.0 or 9.0) at 37 °C. Error bars represent the standard deviation from three biological replicates (*n* = 3).

The essential factor for inhibiting AdiA enzymatic activity at neutral and alkaline conditions was the instability of its oligomeric structure.^[^ ^3^, ^17^ ^]^ The electrostatic repulsion, formed by multiple conserved negatively charged acidic amino acids (e.g., D102, D104, and E467) in the meridional interface between pentameric ring monomers, hindered the oligomerization of decamers (**Figure** **2**a; Figure S1, Supporting Information). Within the dimers, the void formed by the interface interaction of two monomers constituted the substrate channel. Based on prediction with Caver tool, the specific amino acid residues in this channel included T560, V569, P570, T571, R572, T573, Y697, F734, E735, H736, E737, T738 and E739 at A monomer and A158, A159, I192, E193, R194, T195, D202, T204 and G205 at B monomer (Figure 2b). Most channel residues in AdiA from different species sources are highly conserved (Figure S1, Supporting Information). The stability of the oligomerization structure played a decisive role in the formation of substrate channels and active pockets with catalytic functions. Additionally, the acidic amino acids in this channel, including E735, E737, E739, E193, and D202, might contribute to the electrostatic affinity for basic arginine substrates at neutral and alkaline conditions.^[^ ^12^ ^]^ Therefore, substrate channel residues were important targets for engineering because of their dual function in regulating channel translocation efficiency and the strength of interactions between bilayer pentameric rings.

Based on the characterization of the latitudinal and meridional interfaces, a rational cohesion strategy was employed to engineer the pH adaptation of AdiA: i) reducing the electrostatic repulsion within the pentameric ring at neutral and alkaline conditions and ii) improving the affinity of the channel for substrate and the compactness within the dimers.

#### 2.1.1 Rational Engineering of AdiA Decamer Meridional Interface

Based on AdiA catalyzing the conversion of arginine to agmatine, the pH adaptation of AdiA was investigated over the pH 5.5–9.0 range to understand the regulation of the AdiA activity by pH. The optimum pH of AdiA was 5.5, and its enzymatic activity at pH 7.0 was reduced by ≈89% and reduced by ≈98% when pH was ≥ 8.0 (Figure 2c). However, SpeB, agmatinase that catalyzes the synthesis of putrescine from agmatine, had a pH preference for alkaline (Figure S2, Supporting Information), which implies that enhancing the catalytic activity of AdiA under alkaline conditions is necessary to achieve efficient conversion of arginine to putrescine. The high electrostatic repulsion between E467, D102, and D104 was one of the key hindrances to the oligomerization of AdiA decamer. Hence, these acidic amino acids were individually substituted with alkaline amino acids (histidine, arginine, or lysine). Except for the substitutions at site 104, the enzymatic activities of other variants increased at neutral and alkaline conditions (Figure 2c). E467K is better adapted to neutral and alkaline environments compared to other variants. Its specific activity at neutral pH was 3.5‐fold higher than that of AdiA and was 11‐fold higher than that of the wild type at alkaline pH 8.0. Simultaneously, the enzyme activity of E467K was significantly increased under acidic conditions. The lysine substitution at residue 467 was effective in altering the electrostatic interactions and increasing the enzymatic activity of AdiA at neutral and alkaline conditions. The reduced enzymatic activity of all variants at site 104 might be related to their spatial location. D104 was located just at the corner linking the α‐helix to the β‐fold (Figure S3, Supporting Information), whose change tended to disrupt the connection between these two structures, causing a significant change in protein conformation.

#### 2.1.2 Rational Engineering of the AdiA Latitudinal Channel Interface

The nonacidic amino acids in the channel were separately substituted with acidic amino acids (glutamate and aspartate) to increase the affinity of the substrate channel for the alkaline arginine substrate. To rapidly screen for AdiA variants, based on the protons (H^+^) consumption during the arginine decarboxylation catalyzed by AdiA,^[^ ^18^, ^19^ ^]^ a rapid whole‐cell screening method was established. An engineered strain (BL21Δ*3*) was constructed by deleting *the speA* gene encoding constitutive arginine decarboxylase, the *speB* gene encoding agmatinase, and the *adiA* gene. This BL21Δ*3* was used as a host for whole‐cell screening of AdiA variants to eliminate the influence of genomic *speA*, *adiA*, and *speB* on the screening results. In whole‐cell catalytic systems, pH fluctuations during arginine decarboxylation using BL21Δ*3*‐AdiA ranged from 5.4 to 6.25, but no significant fluctuation in pH was found when arginine was converted by BL21Δ*3* (Figure S4a, Supporting Information). Bromocresol purple was used as the pH indicator because its *A*_590_ value was linearly correlated with pH value over the range of pH 5.4–6.8 (Figure S4b, Supporting Information).

After substituting key residues in the substrate channel with acidic amino acids, only the relative enzymatic activities of G205E, H736D, and H736E were increased compared with wild‐type AdiA at pH 6.0 (Figure S5, Supporting Information). The enzymatic activities of most variants decreased, presumably because these substitutions at their locations close to the active center led to conformational changes in the active pocket and enzyme inactivation (Figure 2b). In addition, excessive close aggregation of multiple acidic amino acids on different monomers was detrimental to the oligomerization of dimeric units.^[^ ^12^ ^]^ For example, in R194E, an electrostatic repulsion easily occurred between residues E735, E737, and E739 in monomer A and residues E194 and D202 in monomer B, hindering the oligomerization of monomers A and B (Figure 2b). The specific activities of G205E, H736D, and H736E at different pH values were further analyzed. H736E showed an obvious increase in enzymatic activity at neutral and alkaline conditions, which was 4.6‐fold higher than that of wild‐type AdiA at pH 7.0 (Figure 2c).

#### 2.1.3 Improvement in AdiA Neutral and Alkaline pH Adaptation by Integrated Meridional and Latitudinal Cohesion

As E467K and H736E are respectively located at the latitudinal and meridional interfaces of AdiA, the combined variant E467K_H736E (AdiA‐M2) was designed to improve the AdiA catalytic activity. The pH adaptation of AdiA‐M2 to neutral and alkaline conditions was improved compared with that of E467K and H736E (Figure 2c). The enzyme activity of AdiA‐M2 at neutral pH was 7.6‐fold higher than that of wild‐type AdiA, and at pH 8.0 conditions, it was 35‐fold higher than that of the wild type. AdiA‐M2 still possessed significantly superior catalytic activity at higher alkaline conditions (pH 9.0) (Figure 2c). Furthermore, the enzyme kinetic parameters of AdiA, E467K, H736E, and AdiA‐M2 were measured at pH 7.0. AdiA‐M2 showed the highest *k_cat_* values, which were ≈8.3‐fold higher than those of wild‐type AdiA (**Table** **1**). In E467K, H736E, and AdiA‐M2, the efficiencies of arginine decarboxylation (*k_cat_/K_m_*) at neutral pH were higher than that of AdiA, in which AdiA‐M2 showed an optimal arginine decarboxylation efficiency. In addition, these variants retained the original acid‐tolerant activity of arginine decarboxylase (Figure 2c), which endowed AdiA with optimal whole‐cell catalytic application value, adapting to both acidic environments during the rapid growth of *E. coli* and alkaline environments with high concentrations of putrescine.

**Table 1:** Enzyme kinetic parameters for AdiA and its variants.

Enzyme	AdiA	E467K	H736E	AdiA‐M2
*K*_m_ ± S.E. [mmol·L^−1^]	12.08 ± 0.86	14.30 ± 0.64	15.27 ± 0.79	15.70 ± 0.87
*k*_cat_ ± S.E. [s^−1^]	3.54 ± 0.28	15.19 ± 0.23	17.53 ± 0.55	29.21 ± 0.44
*k*_cat_/*K*_m_ [(mmol·L^−1^)^−1^·s^−1^]	0.29	1.06	1.15	1.86

### 2.2 The Molecular Dynamics Simulations of AdiA and AdiA‐M2

#### 2.2.1 Effect of the Residue Substitution in AdiA‐M2 on the Structure of AdiA Decamers

**Figure 3:**
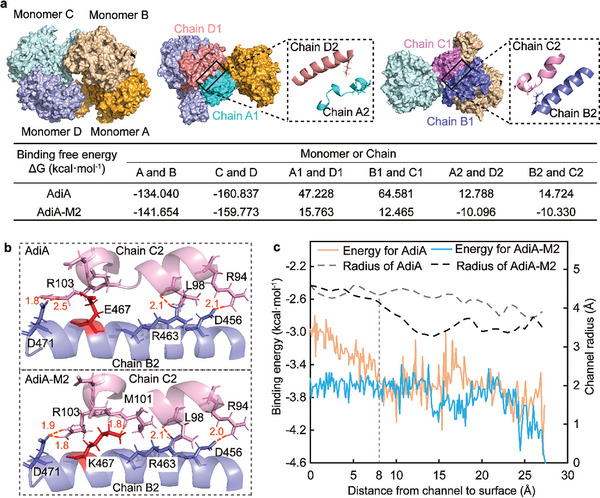
Regulatory mechanism of AdiA variants based on molecular dynamics. a) Binding free energies between different monomers. Monomers A, B, C, and D included all amino acids of the AdiA monomer. Chains A1 and C1 included residues 0–140 of AdiA monomer. Chains B1 and D1 included residues 436–635 of AdiA monomer. Chains A2 and C2 included residues 94–106 of AdiA monomer. Chains B2 and D2 included residues 451–468 of AdiA monomer. b) Interaction between the two interfacial α‐helices located at the interface of the pentameric rings in AdiA and AdiA‐M2. c) Size of the AdiA substrate channel and binding energy of arginine passing through the channel.

Based on molecular dynamics simulations of the AdiA and AdiA‐M2 tetramer at neutrality, the binding free energies (ΔG) between the different monomers or chains were calculated. In the meridian, the ΔG between monomers A and B or C and D in AdiA‐M2 was close to that of AdiA, which was negative showing an affinity (**Figure** **3**a). However, within the latitudinal pentameric ring, the ΔG between chains A1 and D1 or B1 and C1, which contains interfacial residues 102, 104, and 467, was positive in AdiA‐M2 lower than that of AdiA, showing a repulsion (Figure 3a). Moreover, ΔG between the two α‐helices at the pentameric ring interface in AdiA‐M2 (ΔG > 0) was also lower than that in AdiA (ΔG < 0), implying the electrostatic repulsion in AdiA was changed to electrostatic attraction in AdiA‐M2 (Figure 3a). The reduced binding energy between the two α‐helices might be related to the lysine substitution at residue 467. Through bonding interaction analysis, it was observed that the substitution of lysine at residue 467 increased the interaction force between the two α‐helices, forming a new bond between K467 and M101 and shortening the bond length between D471 and R103 (Figure 3b). The enhancement of the interaction between the two α‐helices could provide an additional latitudinal cohesion for AdiA oligomerization, increasing the stability of the AdiA decamer at neutrality.

#### 2.2.2 Effect of Residue Substitution in AdiA‐M2 on the Substrate Channel

Since residue 736 is located at the interface of AdiA dimer (i.e., at the substrate channel) away from the pentamer ring interface, glutamate substitution at residue 736 mainly affected the channel performance and the combination of AdiA dimer. First, the CaverDock tool was employed to characterize the substrate channel (Figure 3c; Figure S6a, Supporting Information). Within 0–8 Å, there is no difference in channel radius between wild‐type AdiA and AdiA‐M2. However, the binding energy of arginine through this part in AdiA‐M2 was lower than that in AdiA, indicating the affinity at the channel entrance to arginine in AdiA‐M2 was increased. Within 8–27 Å, the channel radius in AdiA‐M2 was narrowed, which might be caused by the tighter oligomerization between monomers. The binding energies of substrates through this channel were similar between wild‐type AdiA and AdiA‐M2. Furthermore, based on 3D structure alignment (Figure S6b, Supporting Information), in AdiA‐M2, the orientation of E736 was flipped compared with H736 in AdiA, and the loop with E736 shifts toward the channel center, shortening the distance from T738 on monomer A to D202 on monomer B to 8.9 Å. Meanwhile, the guanidinium group of R194 on monomer B was squeezed toward D202, forming a denser bonding network between monomer A and B (Figure S6c, Supporting Information). The results implied that the substitution of glutamate at residue 736 could reinforce the combination of the dimer (monomers A and B), providing an additional meridional cohesion.

### 2.3 Alkaline Tolerance and Thermal Stability of AdiA and Its Variants

**Figure 4:**
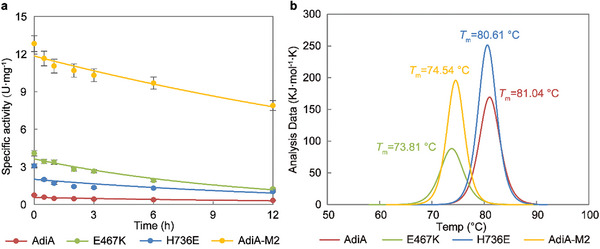
Analysis of alkaline tolerance and thermal stability of AdiA and its variants (E467K, H736E, and AdiA‐M2). a) Tolerance of AdiA and its variants in a pH 8.0 Tris‐HCl buffer at 37 °C. Curves represent the fit for the first‐order exponential decay model. Error bars represent the standard deviation from three biological replicates (*n* = 3). b) Melting temperatures (*T*_m_) of AdiA variants measured by differential scanning calorimeter (DSC).

The downstream products of arginine decarboxylation, agmatine and putrescine, are both alkaline compounds, whose large accumulation leads to a rise in environmental pH to alkalinity. Hence, the alkaline tolerance of AdiA and its variants was explored by incubating in a pH 8.0 Tris‐HCl buffer at 37 °C. AdiA‐M2 is well tolerated to alkalinity, retaining 61% of the enzymatic activity (7.8 U·mg^−1^) for 12 h (**Figure** **4**a). During alkali treatment, the enzyme activity of AdiA‐M2 conformed to a first‐order exponential decay model, with a slower rate of decay (*k* value) than that of the wild type (Table S1, Supporting Information). The good alkaline tolerance of AdiA‐M2 makes it an advantageous candidate for the production of high concentrations of putrescine. Additionally, the thermal stability of AdiA and its variants was analyzed using a differential scanning calorimeter (DSC). The lysine substitution at residue 467 significantly decreased the melting temperatures (*T*_m_) of AdiA (Figure 4b), while the glutamate substitution at residue 736 had less effect on the thermal stability of AdiA. Overall, the thermal stability of AdiA‐M2 was reduced compared with that of wild‐type AdiA. Non‐denaturing gel electrophoresis of AdiA‐M2 showed that AdiA‐M2 with good alkaline tolerance may be attributed to the improved stability of its decameric structure in an alkaline environment (Figure S7, Supporting Information) rather than the improved stability of the monomeric structure.

### 2.4 Putrescine Production Based on Engineered Arginine Decarboxylases

Based on protein engineering, the pH adaptation of three AdiA variants (E467K, H736E, and AdiA‐M2) to neutral and alkaline conditions was significantly improved. These variants were employed to improve the decarboxylation efficiency of arginine in the putrescine synthesis pathway in *E. coli*. The engineered *E. coli* (Δ*EGAP*) deleting the genes associated with putrescine degradation (*speE*, *speG*, and *puuA*) and uptake (*puuP*) was used as a host for producing putrescine. In *E. coli*, besides the inducible AdiA, there is also a constitutive arginine decarboxylase SpeA, which is active as a tetramer with a pH preference at slight alkalinity (pH 7.2–7.4).^[^ ^20^ ^]^ The putrescine produced by constitutive SpeA is involved in the basal physiological activities of cells.^[^ ^21^, ^22^, ^23^ ^]^ The inducible AdiA is mainly involved in the cellular acid stress response to rapidly catalyze arginine decarboxylation to regulate the pH balance between inside and outside of the cell.^[^ ^24^, ^25^, ^26^ ^]^ Here, the putrescine‐engineered strains were optimized by different combinatorial overexpression of key genes *adiA*, *speA*, and *speB*. In shaker fermentation, the strain Δ*EGAP*‐SpeAB‐AdiA, with genes overexpression of *adiA*, *speA* and *speB*, converted 15 g·L^−1^ arginine to synthesize 6 g·L^−1^ putrescine, which increased by 50% compared to that of Δ*EGAP*‐SpeB‐AdiA, with genes overexpression of *adiA* and *speB* (Figure S8a, Supporting Information).

**Figure 5:**
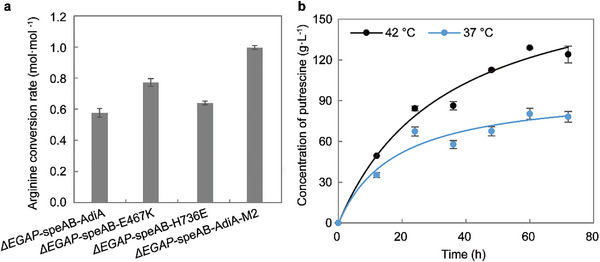
Putrescine production using AdiA and its dominant variants. a) Conversion of arginine to putrescine catalyzed by strains with different AdiA or its variants at the shake flask level. The shake flask was executed in TB medium with 8 g·L^−1^ glucose and 25 g·L^−1^ arginine at 30 °C for 72 h. Error bars represent standard deviation from three biological replicates. b) Whole‐cell catalytic synthesis of putrescine using Δ*EGAP*‐SpeAB‐AdiA‐M2 in pH 7.0 PBS buffer at 37 or 42 °C. Error bars represent the standard deviation from three biological replicates (*n* = 3). Curves represent the fit for the MichaelisMenten equation.

On the basis of Δ*EGAP*‐SpeAB‐AdiA, the synthesis efficiency of putrescine in engineered strain was further optimized with the AdiA mutant (E467K, H736E, or AdiA‐M2). Strains containing AdiA variants demonstrated a higher conversion efficiency in shaker fermentation with 25 g·L^−1^ arginine as substrate. Notably, the molar conversion rate in Δ*EGAP*‐SpeAB‐AdiA‐M2 reached 0.99 mol·mol^−1^ arginine, closely approaching the theoretical value (1 mol·mol^−1^) (**Figure** **5**a). Furthermore, Δ*EGAP*‐SpeAB‐AdiA‐M2 was used for whole‐cell catalytic synthesis of putrescine with 30% ethanol to increase cell permeability and 270 g·L^−1^ arginine as substrate at pH 7.0. The maximal putrescine production reached 128.9 g·L^−1^ after catalyzing for 60 h at 42 °C, which was 1.6 times higher than that at 37 °C (Figure 5b). The molar conversion rate of putrescine was 0.94 mol·mol^−1^ arginine and the production rate was 2.15 g·L^−1^·h^−1^. Production capacity of putrescine per unit cell per unit time reached 0.06 g(product)·g(DCW)^−1^·h^−1^. During whole‐cell catalysis at 42 °C, the system pH reached a maximum of ≈8.0 (Figure S8b, Supporting Information), proving that the selection of AdiA‐M2 with good alkaline adaptation was more suitable. Currently, bio‐based putrescine is mainly synthesized using the ornithine decarboxylase route, and the highest fermentation production reported was 42.3 g·L^−1^ with a molar conversion rate of 0.53 mol·mol^−1^ glucose and production rate of 1.27 g·L^−1^·h^−1^.^[^ ^27^ ^]^ In comparison, whole‐cell bioconversion of putrescine using Δ*EGAP*‐SpeAB‐AdiA‐M2 showed significant advantages in both production and production rate, which was an effective and industrially viable putrescine‐producing process. Additionally, although the raw material for the synthesis of putrescine was arginine in this study, with the development of the arginine biosynthesis process,^[^ ^28^, ^29^, ^30^, ^31^ ^]^ the problem of raw material can be solved by utilizing the co‐culture technique.^[^ ^32^, ^33^ ^]^

### 2.5 Rationally Engineering Neutral pH Adaptation of CadA Based on the Meridional and Latitudinal Cohesion

**Figure 6:**
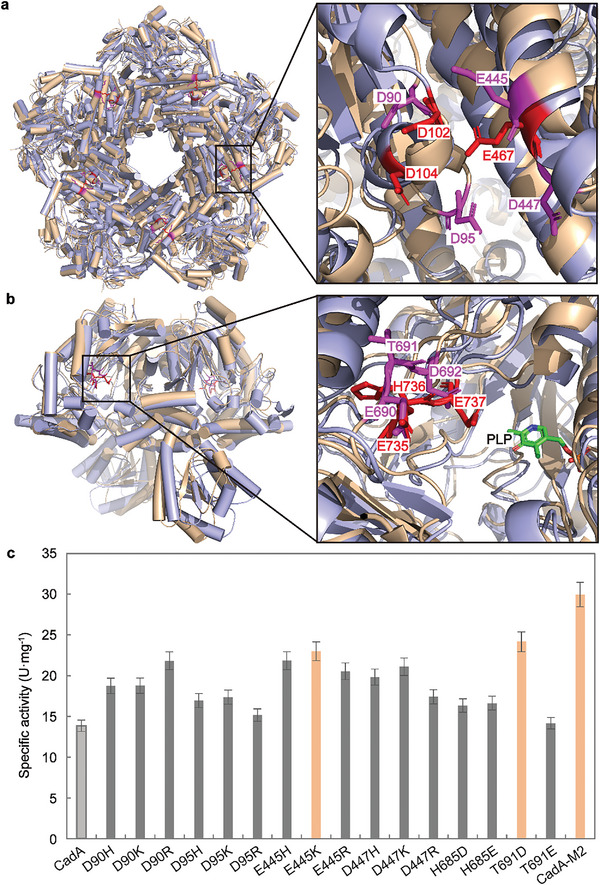
Rational engineering of CadA based on the meridional and latitudinal cohesion strategy. a) Structural alignment of AdiA and CadA (PDB ID:3N75) and key amino acid residues in the pentameric ring of CadA and AdiA. The structure of AdiA is colored purple and the structure of CadA is colored wheat. Key amino acid residues of CadA are colored pink. Key amino acid residues of AdiA are colored red. b) Key amino acid residues in the substrate channel of CadA aligned with AdiA. Key amino acid residues of CadA are colored pink. Key amino acid residues of AdiA are colored red. c) Specific activity of CadA variants engineered at the interaction interface of the pentameric ring and the substrate channel. The error bars indicate the standard deviation of three biological replicates (*n* = 3).

Currently, *E. coli* is used as a chassis cell for the biosynthesis of cadaverine and industrial applications.^[^ ^34^, ^35^, ^36^, ^37^ ^]^ However, the efficiency of cadaverine synthesis is still limited at neutrality due to the inhibition of lysine decarboxylase CadA.^[^ ^3^, ^38^ ^]^ CadA is another important member of the ATR system in *E. coli*, and its active decamer structure is similar to that of AdiA (**Figure** **6**a). Several acidic amino acids are located at the pentameric ring interface of CadA (Asp90, Asp95, Glu445, and Asp447), resulting in strong electrostatic repulsion (Figure 6a), as well as at the entrance of the substrate channel of CadA (Glu690 and Asp692), attracting alkaline lysine substrates (Figure 6b). Moreover, the distribution of these acidic amino acids is consistent with that in AdiA and is also highly conserved in lysine decarboxylases from different sources (Figures S9 and S10, Supporting Information). Hence, the rational engineering of CadA was performed based on the rational cohesion strategy employed for AdiA. Finally, the enzymatic activity of E445K_T691D (CadA‐M2) increased by 2.2‐fold compared with CadA at neutrality (Figure 6c), and CadA‐M2 was well adapted to alkaline pH (Figure S11, Supporting Information). Additionally, in whole‐cell catalysis, the strain containing CadA‐M2 was able to catalyze 2 mol·L^−1^ lysine to synthesize 1.82 mol·L^−1^ cadaverine with a conversion rate of 0.91 mol·mol^−1^, 1.3‐fold higher than that of the wild‐type CadA (Figure S12, Supporting Information). This could potentially address the issue of pH control for the industrial production of cadaverine and the issue of easy deoligomerization for other homotypic decarboxylases at high pH.

## 3 Conclusion

The efficient synthesis of bio‐based putrescine was a milestone for the industrialization of bio‐based nylon 4,6. However, the poor catalytic activity of AdiA at neutral and alkaline conditions posed a challenge to the development of biosynthetic systems for putrescine by whole‐cell catalysis or pure enzymatic catalysis. The enzymatic activity of combination variant E467K_H736E at pH 8.0 increased by 35 folds compared with that of AdiA based on the increase in the cohesion of AdiA decamer and substrate channel turnover. Using this best variant in putrescine synthesis, the putrescine production reached 128.9 g·L^−1^, which was three times higher than that currently reported (42.3 g·L^−1^).^[^ ^27^ ^]^ The conversion of arginine to putrescine was 0.94 mol·mol^−1^, achieving efficient whole‐cell biosynthesis of putrescine. In addition, the rational cohesion strategy could potentially engineer the pH adaptation of the same type of other acid stress‐responsive decarboxylases.

## 4 Experimental Section

### Strains, Media, and Culture Conditions

*E. coli* JM109 cells used as the host strain for DNA manipulation were cultured routinely in Luria‐Bertani (LB) medium with appropriate antibiotics at 37 °C. *E. coli* BL21 (DE3) was used as the starting strain. Putrescine fermentation strains were cultured in a terrific broth (TB) medium with the indicated concentration of arginine and 8 g·L^−1^ glucose. The overexpressed genes were induced by isopropyl β‐D‐1‐thiogalactopyranoside (IPTG) at a final concentration of 1 mmol·L^−1^. Shake‐flask fermentation was performed in 250 mL Erlenmeyer flasks containing 50 mL medium at 37 °C for culturing cells and at 30 °C for inducing gene expression with shaking at 250 rpm. Information for strains and plasmids, and for primers are shown in Tables S2 and S3 (Supporting Information), respectively. Specific molecular assays are detailed in the Supplementary Methods.

### Enzymatic Assays

Enzymatic activities of AdiA and its variants were measured by quantification of agmatine. The system comprised 5 mmol·L^−1^ dithiothreitol (DTT), 0.5 mmol·L^−1^ PLP, 2.5 mmol·L^−1^ MgSO_4_, 20 mmol·L^−1^ arginine, 30 µg·mL^−1^ AdiA or its variants and 1/15 mol·L^−1^ sodium potassium phosphate buffer (pH 5.5, 6.0, 6.5 or 7.0) or Tris‐HCl buffer (pH 8.0 or 9.0). The reaction was carried out at 37 °C in a 200 µL volume for 10 min and was terminated using 40 µL of 40% trichloroacetic acid. The reaction solution was then diluted to determine agmatine concentration. Enzyme kinetic parameters (*K*_m_ and *k*_cat_) of AdiA variants E467K, H736E, and E467K_H736E (AdiA‐M2) were determined using the same system and an arginine substrate concentration range of 0–100 mmol·L^−1^. One specific activity was defined as follows: 1 U·mg^−1^ = 1 µmol of agmatine·min^−1^·mg^−1^ protein. The tolerance of AdiA and its variants to the alkaline environment was investigated by incubating the enzyme in a pH 8.0 buffer at 37 °C various times, followed by an assay of its enzymatic activity.

Whole‐cell specific enzymatic activities of AdiA variants were rapidly assayed by monitoring the absorbance of the whole‐cell catalytic system at a wavelength of 590 nm, using bromocresol purple as an indicator. This system included 90 µL of 0.5 OD_600_ cell fluid from cells overexpressing AdiA or its variants, which were cultured for 16 h in 24‐well plates containing 1 mL of LB medium. The cells were resuspended in pH 5.5 sodium–potassium phosphate buffer, 5 µL of 100 mmol·L^−1^ arginine and 5 µL of 0.1% bromocresol purple. A standard curve of pH was plotted based on the correspondence between different pH (5.5, 6.0, 6.5, and 7.0) and absorbance A_590_ values for the whole‐cell catalytic system without the arginine substrate. Whole‐cell specific enzymatic activities were defined as the ratio of the pH increase per unit time in the whole‐cell catalytic system containing AdiA or its variants relative to that in the system containing wild‐type AdiA.

Enzymatic activities of CadA and its variants were measured using a catalytic system comprising 5 mmol·L^−1^ DTT, 0.5 mmol·L^−1^ PLP, 20 mmol·L^−1^ lysine, 30 µg·mL^−1^ CadA or its variants and 1/15 mol·L^−1^ sodium–potassium phosphate buffer (pH 7.0). The reaction was carried out at 37 °C in a 200 µL volume for 10 min and was terminated using 40 µL of 40% trichloroacetic acid. The reaction solution was diluted to determine cadaverine concentration. One specific activity was defined as follows: 1 U·mg^−1^ = 1 µmol of cadaverine·min^−1^·mg^−1^ protein.

The high‐performance liquid chromatography (HPLC) quantitative assays for agmatine, putrescine, and cadaverine are shown in the Supporting Information.

### Molecular Dynamics Simulations

The structures of AdiA, the best variant AdiA‐M2, PLP, and arginine were cleaned and amended with GaussView 5.0.9 software.^[^ ^39^ ^]^ Molecular dynamics simulations were conducted using the Gromacs 2019.03 package.^[^ ^40^, ^41^ ^]^ The Gromacs editconf program was used to place the tetramer structures of AdiA and the best variant AdiA‐M2, along with PLP and arginine, in an orthorhombic periodic box, ensuring a 10 Å separation margin from the solute in each dimension. The explicit solvation water model SPC216 was placed into the periodic box as the solvent, and Na^+^ ions were added to neutralize the system. The van der Waals and Coulomb potentials were respectively calculated using the simple Cut‐off method and fast smoothing particle mesh PME method. The Amber14SB force field^[^ ^42^ ^]^ for organic molecules was used for protein simulation calculations and the generalized amber force field (GAFF)^[^ ^43^ ^]^ was used for the simulation calculations of PLP and arginine. The antechamber tool of AMBER was used to construct topology and parameters files. All bond lengths involving hydrogen atoms were constrained using the LINCS algorithm.^[^ ^44^ ^]^ The system was first subjected to a single initial steepest‐descent minimization for ≈10 000 cycles. The system was gradually heated from 0 to 298 K in the NVT ensemble for 1 ns, and then relaxed by 1 ns in the NPT ensemble. Finally, 100 ns molecular dynamics simulations were performed for each system.

For binding free energy calculation, 20 frames, with an interval of 1 ns during the 20 ns when the system molecular simulation reached equilibrium, were used to calculate the binding energy in the interaction interface via the MM/PBSA method. All MM/PBSA calculations were performed using a modified gmx_mmpbsa shell script^[^ ^45^ ^]^ and an adaptive Poisson‐Boltzmann solver^[^ ^46^ ^]^ to solve the Poisson‐Boltzmann equation numerically.

The binding energy of the arginine ligand passing through the AdiA substrate channel was analyzed using the Caver Web server (https://loschmidt.chemi.muni.cz/caverweb/). The structures of AdiA and its combination variant obtained by molecular dynamic simulations were used in a Caver Web analysis with arginine as the ligand. Tunnel calculations were performed using Caver 3.02^[^ ^47^ ^]^ by modifying the starting point coordinates at the PLP binding pocket with a maximum distance of 5 Å. After identifying substrate channels, the prepare_receptor4.py script was used to add Gasteiger charges and AutoDock‐Vina^[^ ^48^ ^]^ compatible atom types^[^ ^49^ ^]^ to each atom in the protein structure. The tunneling discretized script^[^ ^50^ ^]^ was then used to cut the tunnel into discrete slices with specified distances. Arginine ligands were processed using the prepare_lignand4.py script and the discretized channels, receptors, and ligands were then forwarded to AutoDock‐Vina and the in‐house tool CaverDock.^[^ ^51^ ^]^ The protein‐ligand binding energy in the discrete channel trajectories was assessed using the fast AutoDock‐Vina algorithm.

### Melting Temperature of Arginine Decarboxylase Detected by DSC

The denaturation temperature (*T*_m_) represents the temperature at which 50% of protein molecules were denatured. DSC was employed comparatively to quantify the stabilizing/destabilizing effects of pH 7.4 Tris‐NaCl buffer. About 1 mg·mL^−1^ protein sample of electrophoretic purity was prepared and dissolved in pH 7.4 Tris‐NaCl buffer. DSC measurements were performed in pH 7.4 Tris‐NaCl buffer using a Nano DSC differential scanning calorimeter (Nano DSC; Waters, Massachusetts, Milford) at a scan rate of 1 °C·min^−1^ from 20 to 100 °C. The sample and reference solution was gently degassed for ≈10 min before loading into the cell. To correct DSC scans, data from relevant controls were subtracted, concentrations were standardized, and *T*_m_ values were fitted and determined using the TwoStateScaled model in NanoAnalyze.

### Whole‐Cell Catalyzed Synthesis of Putrescine Using AdiA‐M2

One positive transformant of strain Δ*EGAP*‐SpeAB‐AdiA‐M2 was inoculated into 25 mL of LB medium and cultured overnight. The cells were then transferred to 50 mL of LB medium at 2% inoculum and cultured for 3 h at 37 °C. Then, 1 mmol·L^−1^ IPTG was added to induce the expression of *adiA* and *speB* genes for 12 h at 30 °C. The fermentation broth was centrifuged at 4 °C and 5000 rpm for 10 min. The supernatant was discarded and the cells were resuspended using PBS buffer at pH 7.0 with 30% ethanol to increase cell permeability. The resuspension was centrifuged again at 4 °C and 5000 rpm for 10 min. The cells were washed three times using PBS buffer at pH 7.0 to remove ethanol, and then the cells were used for whole‐cell catalysis. The whole‐cell catalysis was carried out in a 50 mL centrifuge tube, and the reaction system included: 270 g·L^−1^ arginine adjusted to pH 7.0 with hydrochloric acid, 4 mmol·L^−1^ MgSO_4_, 4 mmol·L^−1^ MnSO_4_, 1 mmol·L^−1^ PLP, 0.4 mmol·L^−1^ DTT, 0.4 mmol·L^−1^ IPTG, 1 mmol·L^−1^ ampicillin, and recombinant cells with a final concentration of OD_600_ = 80. The catalysis system was replenished to 10 mL with PBS buffer at pH 7.0 and was incubated in a constant temperature shaker at 37 or 42 °C and 250 rpm.

### Statistical Analysis

Results presented are mean ± standard error of the mean (SEM) from at least three independent determinations. MichaelisMenten equation and first‐order exponential decay model fitting are performed using Origin 9.1.

## Conflict of Interest

The authors declare no conflict of interest.

## Supporting information

Supporting Information

## Data Availability

The data that support the findings of this study are available from the corresponding author upon reasonable request.
