# Supplementary material for: Rationally Engineering pH Adaptation of Acid‐Induced Arginine Decarboxylase from Escherichia coli to Alkaline Environments to Efficiently Biosynthesize Putrescine
Source: Adv Sci (Weinh). 2024 Apr 3;11(23):2307779. doi: 10.1002/advs.202307779 (PMC11186044; doi:10.1002/advs.202307779)
Supplement: Supplementary file 1 — Supporting Information [file ADVS-11-2307779-s001.pdf]

ADVANCED SCIENCE
Open Access

Supporting Information

for *Adv. Sci.*, DOI 10.1002/advs.202307779

Rationally Engineering pH Adaptation of Acid-Induced Arginine Decarboxylase from *Escherichia coli* to Alkaline Environments to Efficiently Biosynthesize Putrescine

Li Wang, Bo Ding, Xiangyang Hu, Guohui Li and Yu Deng**

Supporting Information

Rationally Engineering pH Adaptation of Acid-Induced Arginine Decarboxylase from *Escherichia coli* to Alkaline Environments to Efficiently Biosynthesize Putrescine

Li Wang,[#] Bo Ding,[#] Xiangyang Hu, Guohui Li, Yu Deng**

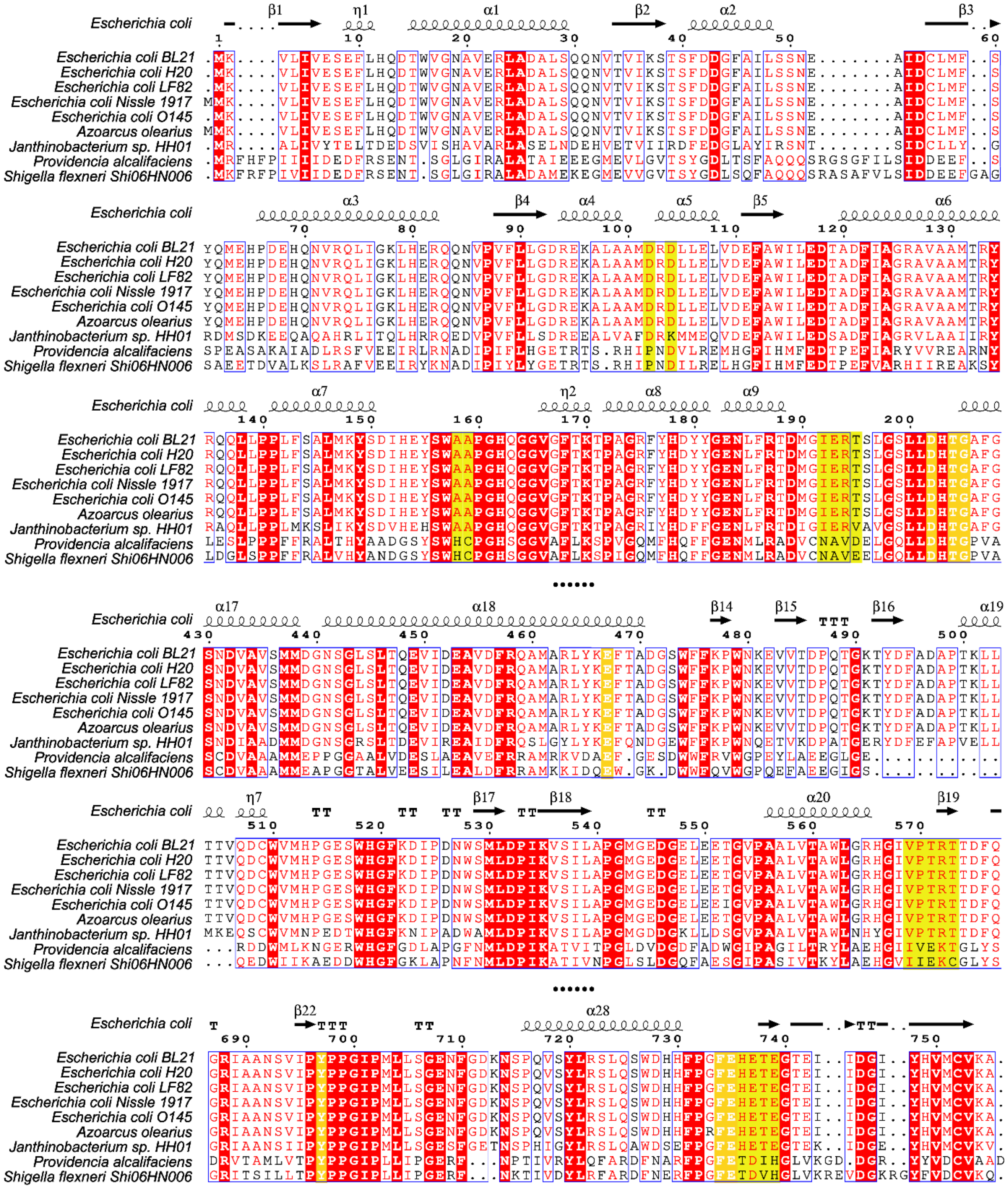

Figure S1. Amino acid residue alignment of AdiAs from different species sources. Key residues at the pentameric ring interface and the channel interface are highlighted in yellow.

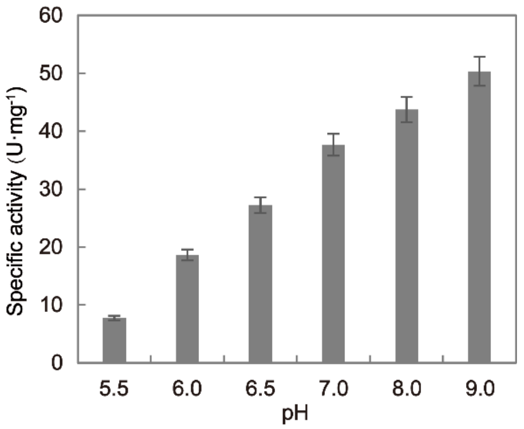

Figure S2. The enzyme activity of SpeB at pH 5.5-9.0.

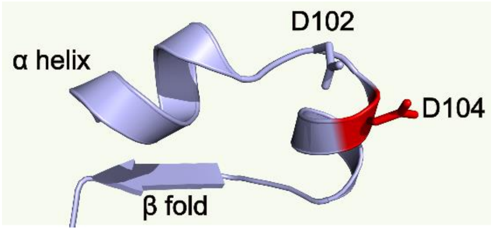

Figure S3. Linking function of residue D104 for α helix and β fold.

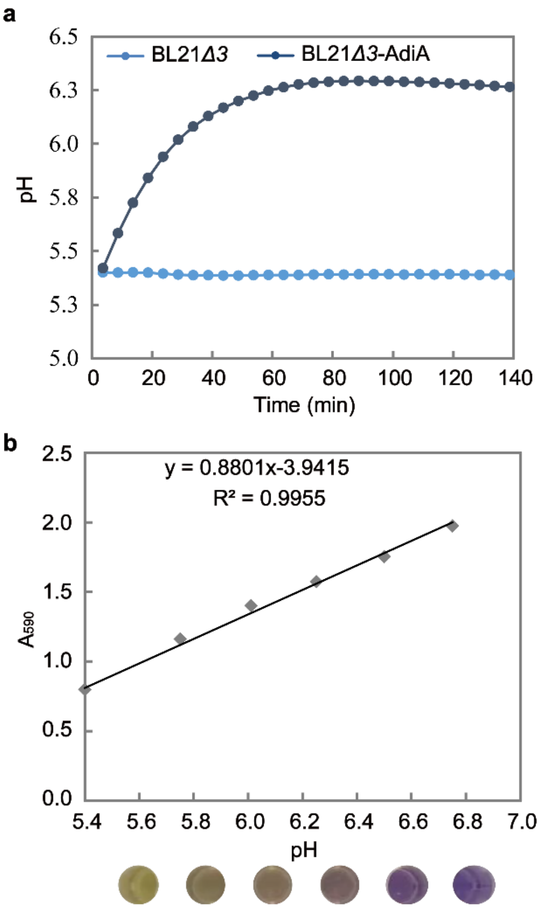

Figure S4. Establishment of a rapid whole-cell screening method for AdiA variants. **a**: Whole-cell catalytic system for arginine at pH 5.4 using strains BL21*Δ3* and BL21*Δ3*-AdiA. BL21*Δ3* was engineered by deleting genes encoding SpeA, SpeB and AdiA. BL21*Δ3*-AdiA

was constructed based on BL21*Δ3* containing pET-adiA. **b**: Standard curves for different pH systems and A_{590} absorbance values with bromocresol purple indicator.

A158D	A158E	A159D	A159E	I192D	I192E	R194D	R194E
0.00	0.00	0.00	0.00	0.00	0.00	0.00	0.00
V569D	V569E	R572D	R572E	Y697D	Y697E	F734D	F734E
0.00	0.00	0.00	0.00	0.00	0.00	0.00	0.00
T571E	T738E	T571D	T195E	P570E	T738D	T560D	P570D
0.16	0.17	0.21	0.25	0.27	0.31	0.43	0.71
T573E	T204D	G205D	T573D	T560E	T195D	T204E	AdiA
0.74	0.75	0.83	0.84	0.84	0.87	0.95	1.00
G205E	H736D	H736E					
1.18	1.29	1.47					
>1	>1	>1					

0 Relative enzyme activity 1.5

Figure S5. Primary screening of AdiA variants with an engineered substrate channel based on relative enzymatic activity. Primary screening experiments were performed in 1/15 mol·L^{-1} sodium potassium phosphate buffer (pH 6.0).

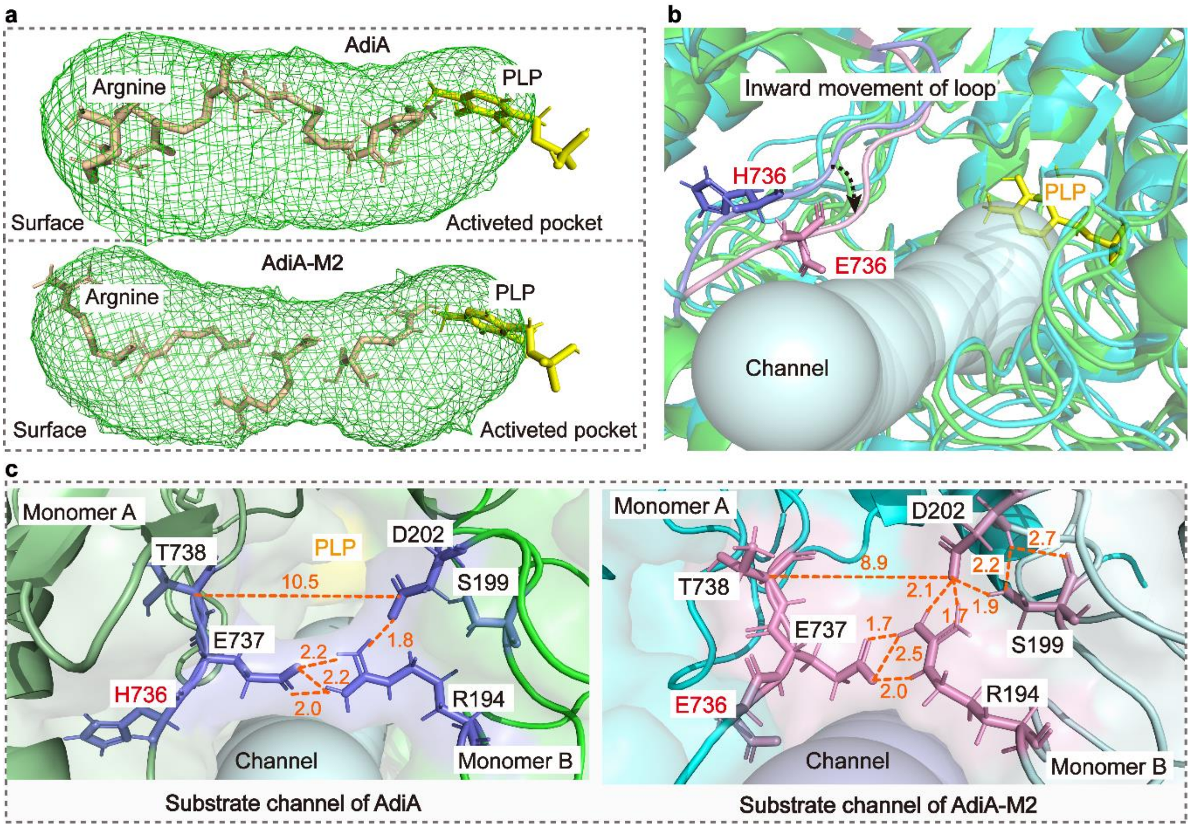

Figure S6. Substrate channel analysis. **a**: CaverDock results for AdiA and AdiA-M2 with arginine. **b**: Structural alignment of substrate channels of AdiA and AdiA-M2. AdiA was colored in green and purple, and AdiA-M2 was colored in bright blue and pink. **c**: Effect of the glutamate substitution at residue 736 on the AdiA substrate channel. Residues H736 (or

E736), E737, and T738 came from monomer A, whereas residues R194, S199, and D202 came from monomer B.

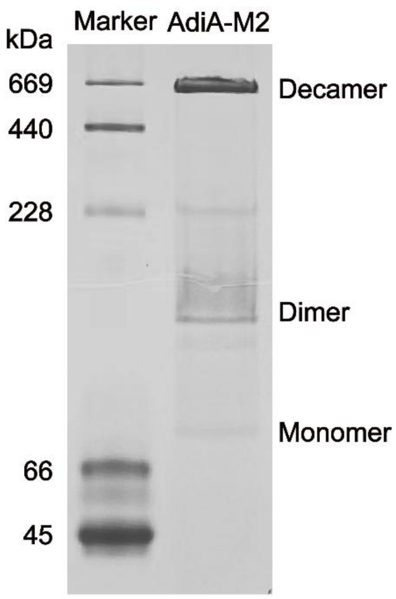

Figure S7. Non-denaturing gel electrophoresis of AdiA-M2. Molecular weight: decamer of 855 kDa, dimer of 171.0 kDa, monomer of 85.5 kDa.

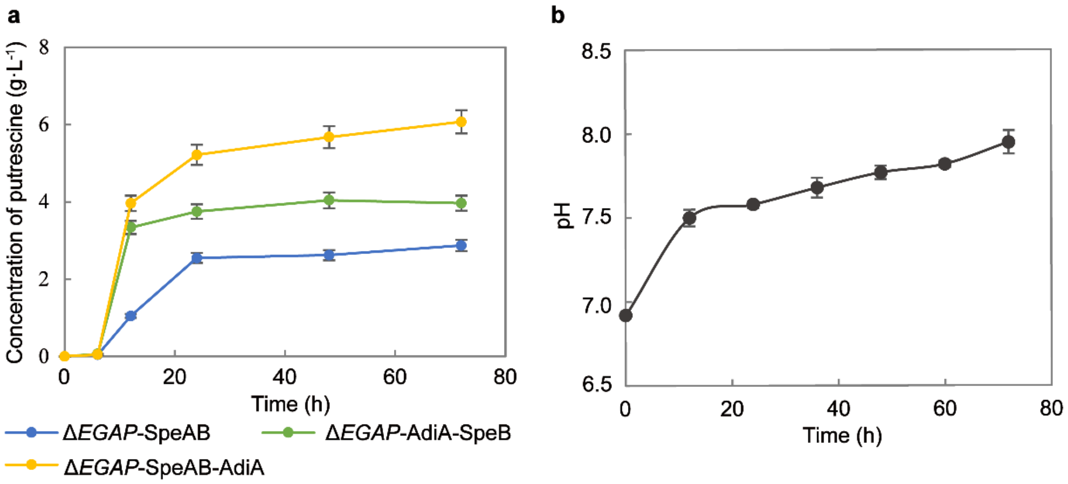

Figure S8. Whole-cell conversion of arginine to synthesize putrescine. **a**: Putrescine synthesis using strains containing different arginine decarboxylase in shake fermentation with 15 g·L^{-1} argnine as substrate. **b**: The pH value during whole-cell catalysis with Δ*EGAP*-SpeAB-AdiA-M2 in pH 7.0 PBS buffer at 42 °C.

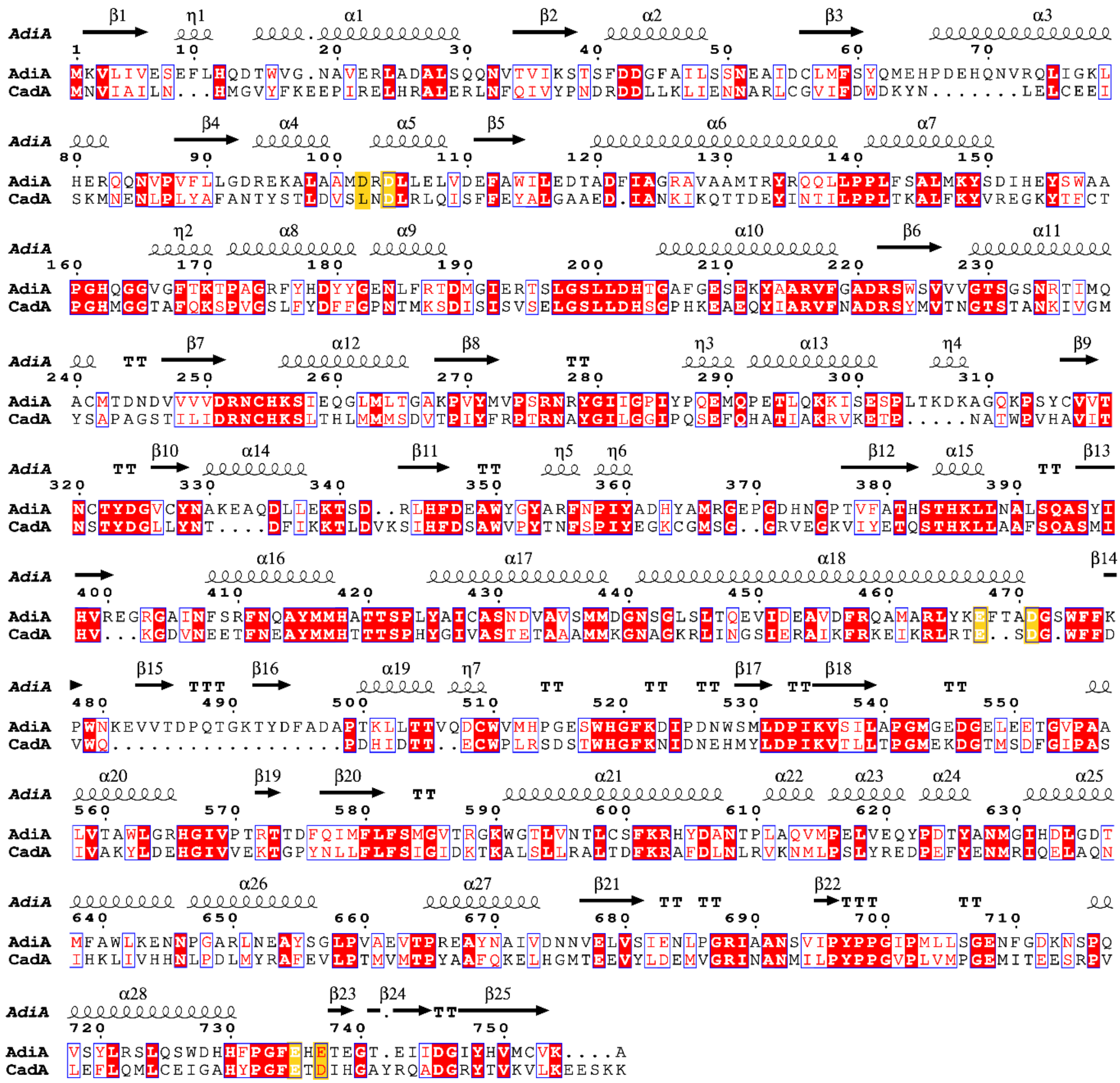

Figure S9. Amino acid residue alignment of AdiA and CadA from *E. coli* BL21. Key acidic amino acid residues are highlighted in yellow.

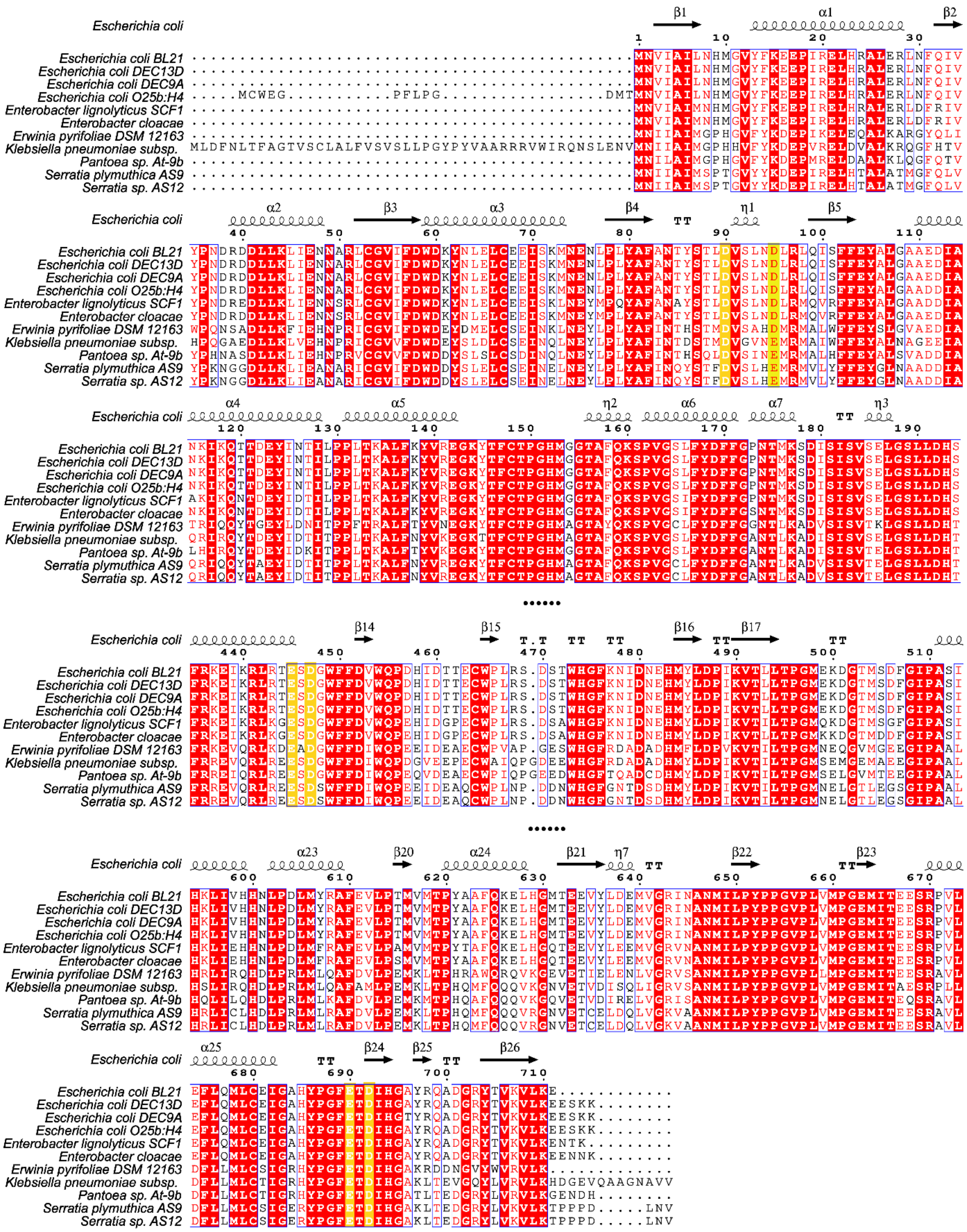

Figure S10. Amino acid residue alignment of CadA from different species sources. Key acidic amino acid residues are highlighted in yellow.

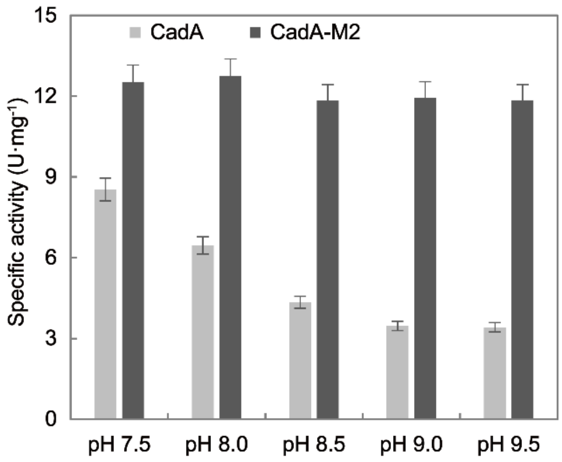

Figure S11. Specific activity of CadA and CadA-M2 at alkaline pH. Enzymatic activity was determined in Tris-HCl buffer (pH 7.5, 8.0, 8.5, 9.0 or 9.5) at 37 °C. The error bars indicate the standard deviation of three biological replicates (n=3).

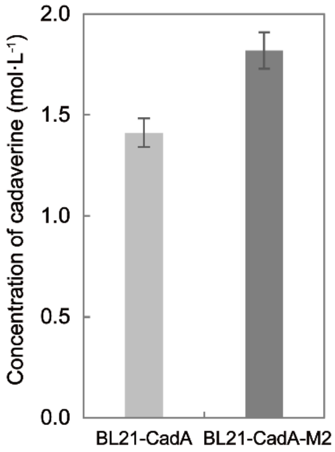

Figure S12. Whole-cell catalytic using engineered strains BL21-CadA and BL21-CadA-M2 overexpressing CadA and CadA-M2 in pH 7.0 PBS buffer at 37 °C. The error bars indicate the standard deviation of three biological replicates (n=3).

Table S1 Fit parameters of first-order exponential decay model $A=A_0e^{-kt}$ for enzyme activity decay

Enzyme	AdiA	E467K	H736E	AdiA-M2
A0±S.E.	0.597±0.051	3.765±0.129	2.258±0.300	11.908±0.282
k±S.E.	0.073±0.026	0.109±0.013	0.107±0.050	0.036±0.006
R^2	0.594	0.952	0.521	0.886

Table S2. Information for strains and plasmids used in this study

**Name**	**Relevant genotype**	**Reference**
**Strains**		
E. coli JM109	For plasmid construction	CICC[a)]
E. coli BL21(DE3)	For expressing genes	CICC[a)]
ΔEGAP	*E. coli* BL21(DE3) deleted genes of SpeE, SpeG, PuuA and PuuP	This study
ΔEGAP-SpeAB	*ΔEGAP* carrying pETDuet-speA-speB	This study
ΔEGAP-SpeB-AdiA	*ΔEGAP* carrying pETDuet-adiA-speB	This study
ΔEGAP-SpeAB-AdiA	*ΔEGAP* carrying pETDuet-speA-adiA-speB	This study
ΔEGAP-SpeAB-E467K	*ΔEGAP* carrying pETDuet-speA-adiA/E467K-speB	This study
ΔEGAP-SpeAB-H736E	*ΔEGAP* carrying pETDuet-speA-adiA/H736E-speB	This study
ΔEGAP-SpeAB-AdiA-M2	*ΔEGAP* carrying pETDuet-speA-adiA-M2-speB	This study
BL21*Δ3*	*E. coli* BL21(DE3) deleted genes of SpeA, SpeB, and AdiA	This study
BL21*Δ3*-AdiA	BL21*Δ3* carrying pET-adiA	This study
BL21-CadA	*E. coli* BL21(DE3) carrying pETDuet-cadA	This study
BL21-CadA-M2	*E. coli* BL21(DE3) carrying pETDuet-cadA-M2	This study
**Plasmids**		
pETDuet-1	ColE1 ori, lacI, T7lac, Amp^R	This study
pETDuet-speA-speB	pETDuet-1 harboring genes *speA* and *speB* from *E. coli* BL21(DE3), Amp^R	This study
pETDuet-adiA-speB	pETDuet-1 harboring genes *adiA* and *speB* from *E. coli* BL21(DE3), Amp^R	This study
pETDuet-speA-adiA-speB	pETDuet-1 harboring genes *speA*, *adiA* and *speB* from *E. coli* BL21(DE3), Amp^R	This study
pETDuet-speA-adiA/E467K-speB	pETDuet-1 harboring genes *speA*, *adiA* variant E467K and *speB* from *E. coli* BL21(DE3), Amp^R	This study
pETDuet-speA-adiA/H736E-speB	pETDuet-1 harboring genes *speA*, *adiA* variant H736E and *speB* from *E. coli* BL21(DE3), Amp^R	This study
pETDuet-speA-adiA-M2-speB	pETDuet-1 harboring genes *speA*, *adiA* variant E467K_H736E (adiA-M2) and *speB* from *E. coli* BL21(DE3), Amp^R	This study
pETDuet-adiA	pETDuet-1 harboring genes *adiA* from *E. coli* BL21(DE3), Amp^R	This study
pETDuet-cadA	pETDuet-1 harboring genes *cadA* from *E. coli* BL21(DE3), Amp^R	This study

[a)] Center of Industrial Culture Collection, Beijing, china. http://cicc.china-cicc.org/.

Table S3. Information for primers used in this study

Primer	Sequence (5' to 3')
speA-F	CATGCCATGGGCTCTGACGACATGTCTATGGGTT
speA-R	CGGAATTCTTACTCATCTTCAAGATAAGTATAACCG
speB-F	GGAATTCGGTACCTAAGGAGATATATCATGAGCACCTTAGGTCATC
speB-R	AAGCTTTTACTCGCCCTTTTTCGC
adiA-F	GGAATTCCATATGAAAGTATTAATTGTTGAAAGCGAGTTTCTCCATCAAGACACCTGGG
adiA-R	CCGCTCGAGTTAATGATGATGATGATGATGCGCTTTCACGCACATAACGT
E467K-F	AAAAAATTCACCGCTGACGGTAGCT
E467K-R	CCGTCAGCGGTGAATTTTTTATATAGCCGCGCCATCG
E467R-F	AAACGTTTCACCGCTGACGGTAGCT
E467R-R	CCGTCAGCGGTGAAACGTTTATATAGCCGCGCCATCG
E467H-F	AAACATTTCACCGCTGACGGTAGCT
E467H-R	CCGTCAGCGGTGAAATGTTTATATAGCCGCGCCATCG
E102K-F	GCAATGAAACGCGACCTGCTGGAGCTTGT
E102K-R	AGGTCGCGTTTCATTGCGGCGAGGGCTTTTTCC
E102R-F	GCAATGCGTCGCGACCTGCTGGAGCTTGT
E102R-R	AGGTCGCGACGCATTGCGGCGAGGGCTTTTTCC
E102H-F	GCAATGCATCGCGACCTGCTGGAGCTTGT
E102H-R	AGGTCGCGATGCATTGCGGCGAGGGCTTTTTCC
E104K-F	AATGGATCGCAAACTGCTGGAGCTTGTCGAT
E104K-R	AGCAGTTTGCGATCCATTGCGGCGAG
E104R-F	AATGGATCGCCGTCTGCTGGAGCTTGTCGAT
E104R-R	AGCAGACGGCGATCCATTGCGGCGAG
E104H-F	AATGGATCGCCATCTGCTGGAGCTTGTCGAT
E104H-R	AGCAGATGGCGATCCATTGCGGCGAG
A158D-F	TGAATATTCCTGGGACGCGCCAGGCCACCAGGGCGGCGTTGGTTTTA
A158D-R	GCGTCCCAGGAATATTCATGGATGTCACTATATTTCATCAGCGC
A158E-F	TGAATATTCCTGGGAAGCGCCAGGCCACCAGGGCGGCGTTGGTTTTA
A158E-R	GCTTCCCAGGAATATTCATGGATGTCACTATATTTCATCAGCGC
A159D-F	TGAATATTCCTGGGCAGACCCAGGCCACCAGGGCGGCGTTGGTTTTA
A159D-R	GTCTGCCCAGGAATATTCATGGATGTCACTATATTTCATCAGCGC
A159E-F	TGAATATTCCTGGGCAGAACCAGGCCACCAGGGCGGCGTTGGTTTTA
A159E-R	TTCTGCCCAGGAATATTCATGGATGTCACTATATTTCATCAGCGC
I192D-F	ATGGGCGACGAACGAACTTCCCTCGGT
I192D-R	AGTTCGTTCGTCGCCCATGTCGGTGCGGAACA
I192E-F	ATGGGCGAAGAACGAACTTCCCTCGGT
I192E-R	AGTTCGTTCTTCGCCCATGTCGGTGCGGAACA
R194D-F	ATGGGCATCGAAGACACTTCCCTCGGTTCTTTG
R194D-R	AGTGTCTTCGATGCCCATGTCGGT
R194E-F	ATGGGCATCGAAGAAACTTCCCTCGGTTCTTTG

Primer	Sequence (5' to 3')
R194E-R	AGTTTCTTCGATGCCCATGTCGGT
T195D-F	ATGGGCATCGAACGAGACTCCCTCGGTTCTTTGCTT
T195D-R	GTCTCGTTCGATGCCCATGT
T195E-F	ATGGGCATCGAACGAGAATCCCTCGGTTCTTTGCTT
T195E-R	TTCTCGTTCGATGCCCATGT
T204D-F	TTGACCATGACGGCGCATTTGGCGAAAG
T204D-R	ATGCGCCGTCATGGTCAAGCAAAGAACCG
T204D-F	TTGACCATGAAGGCGCATTTGGCGAAAG
T204D-R	ATGCGCCTTCATGGTCAAGCAAAGAACCG
G205D-F	TTGACCATACTGACGCATTTGGCGAAAGCGAA
G205D-R	ATGCGTCAGTATGGTCAAGCAAAGAACCG
G205E-F	TTGACCATACTGAAGCATTTGGCGAAAGCGAA
G205E-R	ATGCTTCAGTATGGTCAAGCAAAGAACCG
T560D-F	TGGTCGACGCCTGGCTTGGTCGCCACGGCATTGTA
T560D-R	AAGCCAGGCGTCGACCAGCGCCGCCGGAACACCGGTTTCTT
T560E-F	TGGTCGAAGCCTGGCTTGGTCGCCACGGCATTGTA
T560E-R	AAGCCAGGCTTCGACCAGCGCCGCCGGAACACCGGTTTCTT
V569D-F	ACGGCATTGACCCTACCCGCACCACTGACTT
V569D-R	GTAGGGTCAATGCCGTGGCGACCAAG
V569E-F	ACGGCATTGAACCTACCCGCACCACTGACTT
V569E-R	GTAGGTTCAATGCCGTGGCGACCAAG
P570D-F	ACGGCATTGTAGACACCCGCACCACTGACTT
P570D-R	GTGTCTACAATGCCGTGGCGACCAA
P570E-F	ACGGCATTGTAGAAACCCGCACCACTGACTT
P570E-R	GTTTCTACAATGCCGTGGCGACCAA
T571D-F	ATTGTACCTGACCGCACCACTGACTTCCAAA
T571D-R	TGGTGCGGTCAGGTACAATGCCGTGGCGA
T571E-F	ATTGTACCTGAACGCACCACTGACTTCCAAA
T571E-R	TGGTGCGTTCAGGTACAATGCCGTGGCGA
R572D-F	ATTGTACCTACCGACACCACTGACTTCCAAATTATGTTCCTG
R572D-R	TGGTGTCGGTAGGTACAATGCCGT
R572E-F	ATTGTACCTACCGAAACCACTGACTTCCAAATTATGTTCCTG
R572E-R	TGGTTTCGGTAGGTACAATGCCGT
T573D-F	ATTGTACCTACCCGCGACACTGACTTCCAAATTATGTTCCTGTTCTC
T573D-R	TGTCGCGGGTAGGTACAATGCC
T573E-F	ATTGTACCTACCCGCGAAACTGACTTCCAAATTATGTTCCTGTTCTC
T573E-R	TTTCGCGGGTAGGTACAATGCC
Y697D-F	TCAGTTATCCCGGACCCGCCAGGAATCCCGAT
Y697D-R	CGGGTCCGGGATAACTGAGTTTGCCG
Y697E-F	TCAGTTATCCCGGAACCGCCAGGAATCCCGAT

Primer	Sequence (5' to 3')
Y697E-R	CGGTTCCGGGATAACTGAGTTTGCCG
F734D-F	TCCCTGGAGACGAACACGAAACTGAAGGG
F734D-R	CGTGTTCGTCTCCAGGGAAATGGTGGTC
F734E-F	TCCCTGGAGAAGAACACGAAACTGAAGGG
F734E-R	CGTGTTCTTCTCCAGGGAAATGGTGGTC
H736D-F	TCCCTGGATTTGAAGACGAAACTGAAGGGACTGAAA
H736D-R	CGTCTTCAAATCCAGGGAAATGG
H736E-F	TCCCTGGATTTGAAGAAGAAACTGAAGGGACTGAAA
H736E-R	CTTCTTCAAATCCAGGGAAATGG
T738D-F	CGAAGACGAAGGGACTGAAATTATTGACGG
T738D-R	TCAGTCCCTTCGTCTTCGTGTTCAAATCCAGGGAAATG
T738E-F	CGAAGAAGAAGGGACTGAAATTATTGACGG
T738E-R	TCAGTCCCTTCTTCTTCGTGTTCAAATCCAGGGAAATG
cadA-F	CATGCCATGGGCAACGTTATTGCAATATTGAATCACATGGGGG
cadA-R	CCCAAGCTTTTAATGATGATGATGATGATGTTTTTTGCTTTCTTCTTTCAATACCTTAACGGTATAGCGGCC
D90K-F	TCCACTCTCAAAGTAAGCCTGAATGACCTG
D90K-R	GCTTACTTTGAGAGTGGAATACGTATTAGCG
D90R-F	TCCACTCTCCGTGTAAGCCTGAATGACCTG
D90R-R	GCTTACACGGAGAGTGGAATACGTATTAGCG
D90H-F	TCCACTCTCCACGTAAGCCTGAATGACCTG
D90H-R	GCTTACGTGGAGAGTGGAATACGTATTAGCG
D95K-F	CCTGAATAAACTGCGTTTACAGATTAGCTTC
D95K-R	AAACGCAGTTTATTCAGGCTTACATCGAGAG
D95R-F	CCTGAATCGTCTGCGTTTACAGATTAGCTTC
D95R-R	AAACGCAGACGATTCAGGCTTACATCGAGAG
D95H-F	CCTGAATCACCTGCGTTTACAGATTAGCTTC
D95H-R	AAACGCAGGTGATTCAGGCTTACATCGAGAG
E445K-F	GAACGAAATCTGATGGCTGGTTCTTTG
E445K-R	AGCCATCAGATTTCGTTCTCAGACGTTTGATC
E445R-F	GAACGCGTTCTGATGGCTGGTTCTTTG
E445R-R	AGCCATCAGAACGCGTTCTCAGACGTTTGATC
E445H-F	GAACGCACTCTGATGGCTGGTTCTTTG
E445H-R	AGCCATCAGAGTGCGTTCTCAGACGTTTGATC
E447K-F	GAACGGAATCTAAAGGCTGGTTCTTTGATGTTTG
E447K-R	AGCCTTTAGATTCCGTTCTCAGACG
E447R-F	GAACGGAATCTCGTGGCTGGTTCTTTGATGTTTG
E447R-R	AGCCACGAGATTCCGTTCTCAGACG
E447H-F	GAACGGAATCTCACGGCTGGTTCTTTGATGTTTG
E447H-R	AGCCGTGAGATTCCGTTCTCAGACG
T691D-F	CGGGCTTTGAAGATGATATTCACGGTGCATACCGT

Primer	Sequence (5' to 3')
T691D-R	TATCATCTTCAAAGCCCGGATAGTG
T691E-F	CGGGCTTTGAAGAAGATATTCACGGTGCATACCGT
T691E-R	TATCTTCTTCAAAGCCCGGATAGTG
H694D-F	CCGATATTGATGGTGCATACCGTCAGGCTG
H694D-R	ATGCACCATCAATATCGGTTTCAAAGCCCG
H694E-F	CCGATATTGAAGGTGCATACCGTCAGGCTG
H694E-R	ATGCACCTTCAATATCGGTTTCAAAGCCCG

Supplementary methods

Genes manipulation and plasmid construction: Plasmids used in this study are listed in Table S2. Primers and PCR templates used for cloning genes, constructing plasmids and gene knockout are listed in Table S3. The *speA* gene from *E. coli* BL21 was inserted into the pETDuet-1 using *Nco* I and *Eco*R I restriction endonucleases and T4 ligase to construct plasmid pETDuet-speA. The *speB* gene from *E. coli* BL21 was inserted into pETDuet-speA using *EcoR* I and *Hind* III to construct pETDuet-speA-speB. pETDuet-adiA-speB was constructed using a similar method as pETDuet-speA-speB. The *adiA* gene from *E. coli* BL21 was inserted into pETDuet-speA-speB using *Nde* I and *Xho* I to construct pETDuet-speA-adiA-speB.

To reduce putrescine degradation, key enzyme-encoding genes associated with the putrescine degradation pathway in *E. coli* BL21, including spermidine synthase gene (*speE*),[1] spermidine N-acetyltransferase gene (*speG*),[2] γ-glutamylbutanediamine synthase gene (*puuA*),[3] and putrescine intracellular transporter gene (*puuP*),[4] were deleted using CRISPR-Cas9 technology.[5] Recombinant plasmid pETDuet-speA-speB, pETDuet-adiA-speB, pETDuet-speA-adiA-speB, pETDuet-speA-adiA/E467K-speB, pETDuet-speA-adiA/H736E-speB or pETDuet-speA-adiA-M2-speB was transformed into the *ΔEGAP* knockout strains separately to construct putrescine-producing strains.

The *adiA* gene was inserted into the pETDuet-1 vector via *Nde* I and *Xho* I sites to construct the AdiA overexpression plasmid pETDuet-adiA. The corresponding AdiA variants were constructed basis on pETDuet-adiA using mutagenesis primers listed in Table S3. Vectors with these variants were separately transformed into *E. coli* BL21 for expression. Cells were collected and lysed by a high-pressure cell crusher after culturing in LB medium at 25 °C for 16 h. Proteins were purified using a 5 mL HisTrap HP Ni column.[6] The *cadA* gene from the *E. coli* BL21 genome was inserted into the pETDuet-1 vector via *Nco* I and *Hind* III sites to construct the CadA overexpression plasmid pETDuet-cadA. The expression and purification methods for CadA and its variants were similar to those for AdiA.

Non-denaturing gel electrophoresis: purified AdiA-M2 at a concentration of 800 $\mu g \cdot mL^{-1}$ was used for non-denaturing gel electrophoresis assay with 8% non-denaturing separator gel and 5% non-denaturing concentrate gel. The electrophoresis conditions were 120 V constant voltage for 120 min in electrophoresis buffer at pH 8.0.

Analytical methods: An Agilent 1260 Infinity II high-performance liquid chromatography (HPLC) system (Agilent, Santa Clara, CA, USA) equipped with an Agilent 1260 VWD detector (G7114A) was employed for analysis. An Agilent 1260 vial sampler (G7129A), and

an Agilent 1260 Quat Pump VL (G7111A) were also applied. Analyses were carried out using a Porpshell 120 EC-C18 column (4.6 × 150 mm, 4 μm particle size; Agilent). Quantification of putrescine was achieved by pre-column derivatisation of dansyl chloride as reported previously.[7] Briefly, 3 μL 1,7-heptanediamine (10 mg·mL^{-1}) as internal standard was mixed with 300 μL diluted supernatants of samples. Derivatives were filtered through a 0.22 μm filter membrane for further detection. HPLC separation of dansylated diamines was carried out using the following conditions: column temperature 30 °C, UV detection wavelength 254 nm, injection volume 10 μL, mobile phase solvent A (ultrapure water) and solvent B (acetonitrile). The program for gradient elution was as follows: 0‒4 min, 55‒70% B; 4‒6 min, 70% B; 6‒11 min, 70‒95% B; 11‒12 min, 95% B; 12‒13 min 95‒55% B; 13‒16 min 55% B; flow rate, 0.7 mL·min^{-1}.

Quantification of agmatine was achieved by online derivatization with o-phthaldialdehyde (OPA). The OPA derivatization reagent was prepared by dissolving 10 mg of OPA in 0.5 mL of methanol and mixing with 2 mL of 0.05 mol·L^{-1} borate buffer and 50 μL of β-mercaptoethanol. The samples were derivatized with OPA derivatization reagent using the Agilent 1260 online derivatization feed program and the derivatives were stabilized with 4 μL of 0.1 mol·L^{-1} potassium dihydrogen phosphate solution. HPLC separation procedure was as follows: column temperature 40 °C, UV detection wavelength 338 nm, mobile phase solvent A (pH 7.2, 8 g·L^{-1} sodium acetate solution contained 225 μL triethylamine and 5 mL tetrahydrofuran) and solvent B (6 g sodium acetate dissolved in a mixture of 200 mL of ultrapure water, 400 mL of acetonitrile, and 400 mL of methanol). The program for gradient elution was as follows: 0‒27.5 min, 8‒60% B; 27.5‒31.5 min, 60-100% B; 31.5‒34 min, 100% B; 34‒35.5 min, 100-8% B; 35.5‒45 min 8% B; flow rate, 1 mL·min^{-1}.

Product concentrations were determined using linear regression analysis of peak areas. Calibration curves were prepared using different concentrations of putrescine. The quantification method for cadaverine was the same with that of putrescine.

References

[1] C. W. Tabor, H. Tabor, Q. W. Xie, Proc. Natl. Acad. Sci. U. S. A. 1986, 83 (16), 6040.

[2] J. Fukuchi, K. Kashiwagi, K. Takio, K. Igarashi, J. Biol. Chem. 1994, 269 (36), 22581.

[3] K. Thongbhubate, K. Irie, Y. Sakai, A. Itoh, H. Suzuki, AMB Express 2021, 11 (1), 168.

[4] S. Kurihara, Y. Tsuboi, S. Oda, H. G. Kim, H. Kumagai, H. Suzuki, J. Bacteriol. 2009, 191 (8), 2776.

[5] Y. Jiang, B. Chen, C. Duan, B. Sun, J. Yang, S. Yang, Appl. Environ. Microb. 2015, 81 (7), 2506.

[6] N. Beloglazova, S. Lemak, R. Flick, A. F. Yakunin, Methods Mol. Biol. 2015, 1311, 251.

[7] G. Li, D. Huang, L. Wang, Y. Deng, Biochem. Eng. J. 2021, 166, 107859.
